# Diversity in Leadership at Musculoskeletal Oncology Fellowships in the United States

**DOI:** 10.7759/cureus.35688

**Published:** 2023-03-02

**Authors:** Jason Silvestre, Terry L Thompson, Charles L Nelson, Brock W Adams

**Affiliations:** 1 Orthopedic Surgery, Howard University College of Medicine, Washington, USA; 2 Orthopedics, Perelman School of Medicine, Philadelphia, USA; 3 Orthopedic Surgery, MedStar Washington Hospital Center, Washington, USA

**Keywords:** diversity and inclusion, oncology, orthopedic, surgery, academic, fellowship, leadership

## Abstract

Musculoskeletal oncology fellowship directors (MOFDs) possess the unique ability to influence treatment paradigms in musculoskeletal oncology through teaching and research. Currently, the characteristics that define this important role including demographics, training characteristics, research activity, and grant funding are poorly defined. A list of musculoskeletal oncology fellowship programs was obtained from the American Association of Hip and Knee Surgeons and Musculoskeletal Oncology Fellowship Match. Bibliometric data including the h-index were abstracted from Scopus. Demographics, training, and federal grant characteristics were collected from academic websites. Comparisons were made using t-tests and data were presented as means ± SD. The average age at the appointment was 41±9 years and most were male (80%) and Caucasian (85%). Few had an additional graduate degree (10% MS, 5% PhD). The mean h-index was 23±15 resulting from 91±56 publications. There was a positive correlation between age and h-index (r=0.398, p=0.082). Four MOFDs (20%) had at least one National Institutes of Health research grant. Sex, race, additional graduate degree, and procurement of NIH funding were not associated with higher h-index values. Full professors had higher h-index values than assistant/associate professors (p=0.014). Women and racial minorities are underrepresented among leadership positions in musculoskeletal oncology fellowship programs. This study can help provide a benchmark for departments in orthopedic surgery and aspiring orthopedic surgeons for MOFD positions.

## Introduction

Academic surgeons are promoted based on achievements in research output, clinical productivity, and pedagogy [1]. Orthopedic oncologists refine these skills during orthopedic surgery residency, musculoskeletal oncology fellowship, and early independent practice. Musculoskeletal oncology fellowship directors (MOFDs) possess unique positions of academic influence regarding their ability to train the next generation of musculoskeletal oncologists and create treatment protocols in musculoskeletal oncology.

Currently, the characteristics and accomplishments of MOFD positions are poorly defined. The Accreditation Council for Graduate Medical Education (ACGME) outlines some criteria for MOFDs of ACGME-accredited musculoskeletal oncology fellowships [2]. These include 1.) completion of a musculoskeletal oncology fellowship, 2.) at least three years of independent practice in musculoskeletal oncology, and 3.) at least three years as an academic faculty member. However, benchmarks for promotion into MOFD positions including research activity and grant procurement are poorly described in the literature. Such benchmarks could help aspiring musculoskeletal oncologists prepare for these positions and assist program search committees more effectively recruit MOFDs.

This study elucidates the characteristics of MOFDs in the United States. We asked two questions: 1.) What is the representation of women and underrepresented racial minorities among MOFDs? 2.) What are the academic achievements of MOFDs including academic productivity and federal grant activity? Such data may create a starting point to improve diversity and inclusion efforts at musculoskeletal oncology fellowship programs.

## Materials and methods

This was a retrospective cohort study of MOFDs at musculoskeletal oncology fellowship programs available during the 2021-2022 academic year. An exemption was obtained from the Institutional Review Board given the publicly available nature of all data. Names of musculoskeletal oncology fellowship programs and their respective MOFDs were collected from the American Association of Hip and Knee Surgeons and Musculoskeletal Oncology Fellowship Match. Academic and state medical licensing board websites were queried to obtain demographic and training characteristics for each MOFD. These data included age, gender, race, post-graduate degree, and name of residency and fellowship training programs.

Scopus (Elsevier BV, Waltham, MA) was used to obtain the h-index, number of publications, and number of citations for each MOFD. The h-index is a bibliometric number that is calculated from the maximum number of publications (h) that have been cited at least h number of times for each author. The National Institutes of Health (NIH) REPORT database was used to obtain federal grant procurement data.

Data were presented as means ± standard deviations. Comparisons were made between MOFD characteristics and h-index values with student t-tests with Welch’s correction. H-index values for fellowship directors in other orthopedic sub-specialties were obtained from prior research [3-8]. Correlations between age and h-index values were elucidated via the calculation of the Pearson correlation coefficient. P-values of < 0.05 were considered statistically significant.

## Results

Twenty MOFDs were included in this study (Table 1). All were trained at a U.S.-based orthopedic surgery residency training program. Most were male (80%) and Caucasian (85%). The average age was 49 ± 9 years with an average tenure of 8 ± 9 years as MOFD. MOFDs were appointed to the fellowship program director position at an average age of 41 ± 9 years old.

**Table 1:** Characteristics of musculoskeletal oncology fellowship directors (MOFDs)

Characteristics	Total Number	%
Sex		
Male	16	80
Female	4	20
Race		
Caucasian	17	85
Asian	1	5
Hispanic	0	0
African American	2	10
Age		
30 - 39	2	10
40 - 49	8	40
50 - 59	7	35
60 – 69	2	10
70 - 79	1	5
Post Graduate Degree		
MD only	17	85
MD, PhD	1	5
MD, MSc	1	5
MD, MPH	1	5
Total	20	

15% had an additional graduate degree to an MD. The graduate degrees were MSc (5%), MPH (5%), and PhD (5%). There were no MOFDs with an additional orthopedic surgery clinical fellowship to formal musculoskeletal oncology training.

**Figure 1:**
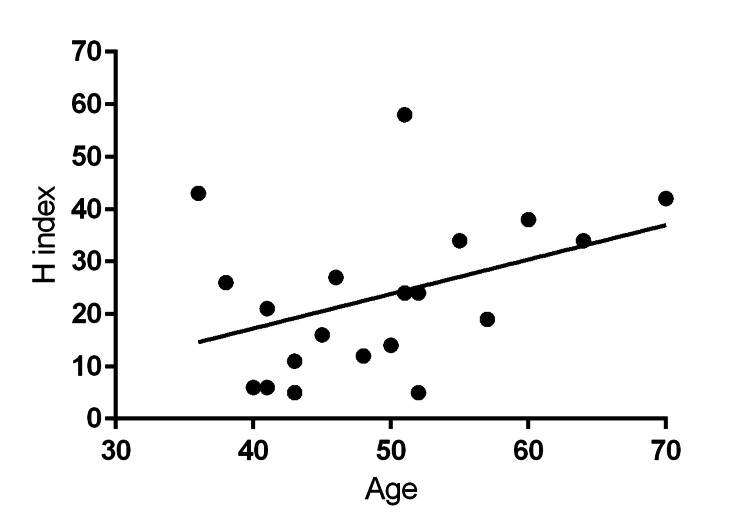
Correlation of H-index and age of musculoskeletal oncology fellowship directors (MOFDs) *Pearson coefficient, r = 0.398, p = 0.0821

The mean h-index was 23 ± 15. MOFDs had a mean of 91 ± 56 publications that were cited 2,586 ± 2,947 times. The top 10 MOFDs by h-index are listed in Table 2. There was a positive correlation between age and h-index (r = 0.398, Figure 1), but this did not reach statistical significance (p = 0.0821). 55% of MOFDs were trained by the top five represented musculoskeletal oncology fellowships compared with 30% of the top five represented orthopedic surgery residencies (p = 0.200).

**Table 2:** Top musculoskeletal oncology fellowship directors (MOFDs) as measured by the H-index

Musculoskeletal Oncology Fellowship Program	H-index
University of Chicago	58
University of Washington	43
University of Texas Houston	42
University of Toronto	38
University of Florida	34
University of Texas MD Anderson Cancer Center	34
University of Utah/Huntsman Cancer Institute	27
Mayo Clinic Minnesota	26
Massachusetts General Hospital	24
Vanderbilt University Medical Center	24

Four MOFDs (20%) had at least one NIH research grant for a total of seven grants (Table 3). The National Cancer Institute (NCI, 90.3%) and National Institute for Arthritis and Musculoskeletal and Skin Diseases (NIAMS, 9.7%) awarded this funding. The T32, K08, P01, R01, K08, and U54 grant mechanisms were represented.

**Table 3:** National Institutes of Health (NIH) funding awarded to musculoskeletal oncology fellowship directors (MOFDs) NIAMS = National Institute for Arthritis and Musculoskeletal and Skin Diseases; NCI = National Cancer Institute; total funding refers to total costs awarded from the 1996 – 2021 over the course of a MOFD’s career

Institute	Mechanism	Total NIH Funding ($)	% of total	No. of grants
NCI	U54	11,520,799	71.2	1
	R01	1,744,220	10.8	1
	K08	896,400	5.5	1
	P01	448,990	2.8	1
NIAMS	K08	1,298,905	8.0	2
	T32	279,174	1.7	1
Total	-	16,188,488	100	7

Predictors of higher MOFD h-index values are displayed in Table 4. Sex, race, additional graduate degree, and procurement of NIH funding were not associated with higher h-index values (p > 0.05, Table 4). Full professors had higher h-index values than assistant/associate professors (37 ± 17 vs 20 ± 13, p = 0.014). When compared with fellowship directors from other orthopedic subspecialties, musculoskeletal oncologists ranked among the top two for academic productivity (Table 5).

**Table 4:** Characteristics associated with academic productivity of musculoskeletal oncology fellowship directors (MOFDs) *Additional graduate degrees include MPH, MSc, and PhD; Student’s t-test with Welch’s correction gives p < 0.05

Characteristics	N	Mean h-index ± SD	P
Sex			0.675
Female	4	21 ± 10	
Male	16	24 ± 16	
Race			0.482
Non-Caucasian	3	17 ± 15	
Caucasian	17	24 ± 15	
Graduate Degree*			0.513
Yes	3	34 ± 27	
No	17	21 ± 12	
Academic Rank			0.014*
Assistant / Associate	11	16 ± 12	
Full Professor	9	32 ± 14	
NIH Funding			0.126
Yes	4	37 ± 17	
No	16	20 ± 13	

**Table 5:** Summary of fellowship directors across orthopedic subspecialties

Specialty	Authors	Year Published	No of FDs	Mean age	Mean H-index
Spine Surgery	Donnally et al.	2020	103	53	24
Oncology	Silvestre et al.	2022	20	55	23
Sports Medicine	Schiller et al.	2021	82	56	23
Pediatric	Cohen et al.	2021	55	51	17
Adult Reconstruction	Schiller et al.	2020	94	53	17
Trauma	Sama et al.	2021	72	51	15
Foot and Ankle	Elahi et al.	2021	47	51	13

## Discussion

MOFDs have strong research qualifications including research output as measured by the h-index. A minority of MOFDs had NIH grants funding (20%) and this achievement was not associated with higher h-index values. Overall, women and racial minorities are underrepresented among leadership positions in musculoskeletal oncology training programs. Interestingly, the only characteristic associated with higher h-index values was promoted to full professor rank, reinforcing its utility as a barometer for academic progression. MOFDs had the second-highest h-index values across fellowship directors of orthopedic subspecialties.

Results from this study help to establish a benchmark for academic achievements a musculoskeletal oncologist must possess to get promoted to the MOFD position. On average, MOFDs get promoted to this position at 41 years of age. The average h-index was 23, which is higher than other reported benchmarks of academic orthopedic surgeons [9-13]. A study of 2,061 orthopedic surgery faculty from 120 residency programs found an average h-index of 15 for full professors and 18 for chairs [9]. The value of highly productive leaders in departments of orthopedic surgery has been described previously [12]. Program directors and chairs set strategic priorities of departments and help develop research infrastructures to produce manuscripts and obtain grants. While MOFDs are incredibly accomplished academically, future studies should also assess qualitative attributes like leadership ability and emotional intelligence of these leaders.

Overall, the lack of gender and ethnic diversity is a well-described challenge in the field of orthopedic surgery [14-18]. There has been renewed interest in spurring inclusion and diversity initiatives across orthopedic surgery including among the most junior ranks [15]. Change among leadership positions in academia will be gradual as the pipeline of under-represented minorities matures. Similar needs exist within musculoskeletal oncology, which parallels those in medical oncology [19]. Ultimately, increasing the gender and racial diversity of MOFDs s is important given the high visibility of these leaders and their influence over future generations of musculoskeletal oncologists. Diversity and inclusion efforts at senior leadership levels are self-reinforcing as under-represented minority medical students and residents develop mentorship relationships with these leaders [20]. More research is needed to understand creative ways to increase the pipeline of diverse talent to promote more women and racial minorities into senior leadership positions in academic orthopedics.

NIH-funded surgeon scientists are an endangered species [21-23]. Decreasing funding levels awarded to surgeon scientists and barriers to NIH grant procurement in academic orthopedic surgery have been well-described [24,25]. In our study, 20% of MOFDs received at least one NIH research grant. Extrapolating this finding to the entire field of musculoskeletal oncology was limited by our small sample size. More research is needed to understand the entire portfolio of NIH funding across musculoskeletal oncology and understand potential barriers to successful grant procurement. While funding levels have stagnated and even decreased over time when adjusted for inflation, NIH research grants remain an important source of biomedical research funding and innovation in the United States [23].

There were several limitations stemming from the retrospective nature of this study. At best, this study creates a snapshot of qualifications for MOFDs as of the 2021-2022 academic year. It was not possible to obtain temporal trends over time given the cross-sectional design. Furthermore, several key demographic and academic attributes were analyzed in this study. Other intangible criteria like pedagogical capacity, leadership ability, and administrative skills were not captured. Operative volume was also not accessible, which has been identified as an important metric for advancement in academic surgery. Lastly, given the small sample size of MOFDs available for study, our study was likely underpowered to discover all associations with great accuracy. For example, while there was a positive correlation between age and h-index in our study, this finding did not reach statistical significance.

## Conclusions

In conclusion, MOFDs achieve high levels of scholarly output when compared with the accomplishments of colleagues from other orthopedic subspecialties. MOFDs are largely male and Caucasian, which may highlight a need for greater diversity and inclusion initiatives in the field of musculoskeletal oncology. There is a large degree of institutional variability in MOFD accomplishments given the lack of standardized selection criteria for these positions. Ultimately, however, results from this study can be used as starting point for these leadership positions and a baseline to improve diversity and inclusion efforts in the field of musculoskeletal oncology.
